# Burnout, daytime sleepiness and sleep quality among technical-level Nursing students*

**DOI:** 10.1590/1518-8345.5180.3487

**Published:** 2021-10-29

**Authors:** Kawanna Vidotti Amaral, Maria José Quina Galdino, Júlia Trevisan Martins

**Affiliations:** 1 Universidade Estadual de Londrina, Londrina, PR, Brazil.; 2 Universidade Estadual do Norte do Paraná, Departamento de Enfermagem, Bandeirantes, PR, Brazil.

**Keywords:** Disorders of Excessive Somnolence, Psychological Burnout, Nursing Students, Associate Nursing Education, Sleep, Sleep Deprivation, Trastornos de Somnolencia Excesiva, Agotamiento Psicológico, Estudiantes de Enfermería, Graduación en Auxiliar de Enfermería, Sueño, Privación de Sueño, Distúrbios do Sono por Sonolência Excessiva, Esgotamento Psicológico, Estudantes de Enfermagem, Educação Técnica em Enfermagem, Privação do Sono, Sono

## Abstract

**Objective:**

to evaluate the association of the burnout syndrome with daytime sleepiness and sleep quality among technical-level Nursing students.

**Method:**

a cross-sectional, analytical and quantitative study, conducted with 213 students from four technical Nursing courses in a city of Paraná, Brazil. Data collection was carried out using an instrument containing characterization information, the Maslach Burnout Inventory - Student Survey, the Epworth Sleepiness Scale and the Pittsburgh Sleep Quality Index. The data were analyzed using descriptive statistics and logistic regression.

**Results:**

the prevalence values of the burnout syndrome, excessive daytime sleepiness and poor sleep quality were 4.7%, 34.7% and 58.7%, respectively. Excessive daytime sleepiness significantly increased the chances of high emotional exhaustion (OR^adj^: 5.714; p<0.001) and high depersonalization (OR^adj^: 4.259; p<0.001). Poor sleep quality, especially sleep disorders, was associated with all dimensions of the syndrome (p<0.05).

**Conclusion:**

high levels of the burnout syndrome dimensions were associated with excessive daytime sleepiness and poor sleep quality. Educational institutions should include sleep hygiene and psychosocial support in their student health promotion programs.

## Abstract (es)

**Objetivo:**

evaluar la asociación del síndrome de *burnout* con la somnolencia diurna y la calidad del sueño en estudiantes de enfermería de nivel técnico.

**Método:**

estudio transversal, analítico y cuantitativo, con 213 estudiantes de cuatro carreras técnicas de enfermería en una ciudad del estado de Paraná, Brasil. La recolección de datos se realizó utilizando un instrumento que contiene información de caracterización, el *Maslach Burnout Inventory - Student Survey*, la Escala de Somnolencia de Epworth y el Índice de Calidad del Sueño de Pittsburgh. Los datos se analizaron mediante estadística descriptiva y regresión logística.

**Resultados:**

las prevalencias del síndrome de *burnout*, de la somnolencia diurna excesiva y de la mala calidad del sueño fueron 4,7%, 34,7% y 58,7%, respectivamente. La somnolencia diurna excesiva aumentó significativamente las posibilidades de alto agotamiento emocional (OR^aj^: 5,714; p<0,001) y alta despersonalización (OR^aj^: 4,259; p<0,001). La mala calidad del sueño, especialmente las alteraciones del sueño, se asoció con todas las dimensiones del síndrome (p<0,05).

**Conclusión:**

altos niveles de las dimensiones del síndrome de *burnout* se asociaron con somnolencia diurna excesiva y mala calidad del sueño. Las instituciones educativas deben incluir la higiene del sueño y el apoyo psicosocial en sus programas de promoción de la salud de los estudiantes.

## Abstract (pt)

**Objetivo:**

avaliar a associação da síndrome de *burnout* com a sonolência diurna e a qualidade do sono entre alunos de nível técnico em enfermagem.

**Método:**

estudo transversal, analítico e quantitativo, com 213 alunos de quatro cursos técnicos em enfermagem de uma cidade do Paraná, Brasil. A coleta de dados foi realizada por meio de um instrumento contendo informações de caracterização, o *Maslach Burnout Inventory - Student Survey*, a Escala de Sonolência de Epworth e o Índice da Qualidade do Sono de Pittsburgh. Os dados foram analisados por estatística descritiva e regressão logística.

**Resultados:**

as prevalências da síndrome de *burnout,* da sonolência diurna excessiva e da qualidade do sono ruim foram 4,7%, 34,7% e 58,7%, respectivamente. A sonolência diurna excessiva aumentou significativamente as chances de alta exaustão emocional (OR^aj^: 5,714; p<0,001) e alta despersonalização (OR^aj^: 4,259; p<0,001). A qualidade do sono ruim, sobretudo os distúrbios do sono, associou-se com todas as dimensões da síndrome (p<0,05).

**Conclusão:**

altos níveis das dimensões da síndrome de *burnout* foram associados à sonolência diurna excessiva e à má qualidade de sono. As instituições de ensino devem incluir a higiene do sono e o apoio psicossocial em seus programas de promoção à saúde dos estudantes.

## Introduction

Worldwide, burnout syndrome^(1)^ and sleep problems^(2)^ among students have been indicated as a public health concern, due to the repercussions on learning and on the biopsychosocial-spiritual health of these individuals^(3)^. Students in the health area and, especially, in Nursing, are very vulnerable due to the characteristics of their training, permeated by conflicts with colleagues and professors, concerns and difficulties in acquiring the necessary knowledge to become good professionals, enter the labor market and provide direct assistance to the patients, who may be in distress^(4-5)^.

The burnout syndrome in students is understood as a three-dimensional process that involves: emotional exhaustion, expressed by the feeling of being exhausted with the study loads; depersonalization, with attitudes of distancing from school activities and reduced academic efficacy, defined by the self-perception of not having competence for studying^(6)^.

The syndrome arises from the chronicity of stress, exhaustion and insufficient resources to deal with both^(6)^; sleep complaints are considered an additional symptom that frequently occurs in this emotional disorder^(3)^. A number of studies showed that, among university students, sleep quality is indirectly related to the syndrome^(3,7-8)^.

Sleep is a human health behavior, with a role in various systems, especially immune, as well as in the metabolism, cognition and emotional regulation^(9)^. Due to its restoring power for the body and mind^(9)^, sleep quality involves the individual’s perception of rest and sleep depth^(10)^. Its deprivation increases the propensity for excessive daytime sleepiness, characterized by falling asleep at inappropriate times, as well as reflecting the inability to promptly respond to external and concentration stimuli^(11)^.

From this perspective, sleep problems in students impair their learning ability, reduce academic performance and increase the occurrence of chronic health conditions^(12)^. Such aspects exert an impact on the training of people for qualified health care to the population, especially nursing technicians who, together with nursing assistants, comprise nearly half of the health workforce in Brazil^(13)^.

In the training of health students, especially nursing technicians, there can be several stressors such as: high workload, which needs to be reconciled with work activities, excessive amount of evaluative activities, power relationship between professors and students and professional practical classes in the health services, in which fear of making harmful errors for the patient’s life is imminent^(14)^. To meet the demands and achieve good academic performance, the students can relegate their personal life, sleep and rest.

Given the considerations presented and the lack of studies relating to the burnout syndrome with sleep quality and excessive daytime sleepiness among students of technical Nursing courses, research studies with this objective, in addition to advancing knowledge, may be strongholds for planning actions aimed at promoting health and preventing health problems in this population and, consequently, provide more favorable conditions in the teaching-learning process.

The hypothesis advocated in this study is that excessive daytime sleepiness and poor sleep quality are directly associated with the burnout syndrome dimensions, regardless of gender, age and exercising work activities in conjunction with studies and depression.

Such being the case, the objective was to evaluate the association of the burnout syndrome with daytime sleepiness and sleep quality among technical-level Nursing students.

## Method

### Study design

This is a cross-sectional and analytical study with a quantitative approach. Such being the case, the description of this section followed the Strengthening the Reporting of Observational Studies in Epidemiology (STROBE) guideline.

### Locus and data collection period

The study was carried out between March and June 2020 in all the Nursing technician courses in the city of Londrina, Paraná (PR), Brazil. Two courses were public and two private, offering classes during the day and at night, with hour loads ranging between 1,200 and 1,800 hours, distributed over four semesters.

### Population, sample and selection criteria

The target population was represented by 560 eligible students from the aforementioned Nursing technician courses, who met the following criteria: being 18 years old or over, not being away from the course activities due to leaves of any nature and being enrolled in the last two semesters, since the hour loads are identical and the professional practical classes take place in the four courses under study.

All the eligible students were invited and the sample consisted of 213 students, therefore being a convenience sample. This number of participants was considered adequate in relation to the proposed statistical analysis, which requires a minimum of 10 cases for each variable inserted in the multiple model, with a minimum sample of 100 cases^(15)^.

### Study variables and instruments

For data collection, the authors developed an instrument that contained sociodemographic, occupational, academic and living conditions: age (in years old), gender (female or male), marital status (single, married/stable union, widowed, separated/divorced or other marital status), with whom they live (alone, family, colleagues or others), exercise of work activity (no or yes), type of technical course (public or private), semester attended (penultimate or last) and use of antidepressants (no or yes).

The burnout syndrome was assessed using the Brazilian version of the Maslach Burnout Inventory - Student Survey (MBI-SS), which was duly adapted and validated^(16-17)^. It is a self-administered questionnaire composed of 15 items assessing three conceptual dimensions: emotional exhaustion, depersonalization and academic efficacy, in which the answers have a Likert-type scale format (0-6)^(14)^. The scale does not generate an overall score, but the scores can be dichotomized into high emotional exhaustion (≥16 points), high depersonalization (≥11 points) and low academic efficacy (≤23 points)^(6)^. High levels of emotional exhaustion and depersonalization and low academic efficacy indicate the burnout syndrome. This instrument was previously used to assess this syndrome among Nursing technical course students^(14)^, even by the authors themselves, who carried out the translation and verification of the psychometric properties of MBI-SS^(17)^. Such being the case, it was considered the appropriate instrument, since the statements refer to how the students relate to their studies.

Sleep quality was verified by means of the Pittsburgh Sleep Quality Index - PSQI, its version translated for Brazil, with good internal consistency and factorial validity. It is a self-administered scale with 13 questions which, after transformation^(10)^, result in seven components from 0 to 3 points: subjective sleep quality, sleep latency, sleep duration, habitual sleep efficiency, sleep disorders, use of sleep medication and daytime dysfunction. The sum of these items varies from 0 to 21 points and classifies this result as good sleep quality (0-4 points), poor sleep quality (5-10 points) and sleep disorder (10-21 points)^(10)^.

In its adapted and validated version for the written/spoken Brazilian Portuguese, the Epworth Sleepiness Scale (ESS) was used to assess excessive daytime sleepiness. This questionnaire assesses the probability of falling asleep during the day in eight situations involving daily activities (sitting and reading; watching television; sitting quietly in a public place; sitting for an hour as a passenger in a car; lying down in the afternoon to rest; sitting and talking to another person; sitting quietly after lunch without drinking alcohol; sitting in a car parked in traffic for a few minutes), whose alternatives are provided in Likert-type scales (from 0 to 3 points). The overall score varies from 0 to 24, with scores ≥10 being considered excessive daytime sleepiness^(11)^.

It is noteworthy that PSQI and ESS assess the sleep quality and daytime sleepiness of any individual, as they do not include questions aimed at specific groups. In this sense, a number of national and international scientific studies^(18-22)^ have used these instruments to verify such constructs in students.

### Data collection

Given the covid-19 pandemic and the fact that the educational institutions do not recommend carrying out in-person activities involving agglomeration of people, it was decided to conduct data collection via Internet. Such being the case, the research instrument was included in the Google Forms platform, with all the variables to be filled out and in the following order: characterization questionnaire, PSQI, ESS and MBI-SS, each separated by screens, which the participant accessed, answered and, at the end, clicked on the word “next”, thus constituting a single file.

Considering that the characterization questionnaire was not previously validated, it was decided to carry out a pilot test with the complete data collection instrument with 30 students from a technical Nursing school in a neighboring city with characteristics similar to those of the study population to verify ambiguities, understanding of the issues and operationalization of collection.

As it was not necessary to make adjustments, between March and June 2020 all the students were invited to participate in the study, sending the link to the study instrument to their personal email addresses (provided by the course coordinators). It is to be noted that the scale questions were not modified, that there is no restriction regarding the application format (paper, cell phones and computers, among others) and that its application over the Internet was used to verify the cross-cultural validity of MBI-SS^(16)^.

Access to the data collection questionnaire was preceded by acceptance to participate in the research through the Informed Consent Form (ICF). The answers were automatically directed to a database.

### Data treatment and analysis

The data were analyzed using the Statistical Package for the Social Sciences (SPSS), version 20.0. In descriptive statistics, the data were presented in absolute and relative frequencies and the “age” quantitative variable was presented as median and interquartile range (IRQ), considering that the Shapiro-Wilk test indicated non-adherence to normal distribution (p<0.001).

Cronbach’s alpha was calculated in order to verify the reliability of the scales used in this study. The dependent variables were the burnout syndrome dimensions, high emotional exhaustion, high depersonalization and low academic efficacy. The independent variables were excessive daytime sleepiness (dichotomous category), sleep quality (dichotomous category), and the PSQI components (numerical).

The association of outcomes and exposures was verified by univariate binary logistic regression. Subsequently, multiple logistic regressions were performed to adjust the association, considering the “gender”, “age” (in years old), “working” and “use of antidepressants” variables, for being aspects that can be potential confusing factors for the relationship^(23)^. The model’s fit was verified by the Hosmer-Lemeshow test and the variation was explained by Nagelkerke R Square. The results were presented in *odds ratio* with 95% confidence interval (95% CI), that is, α<0.05 was considered statistically significant.

### Ethical aspects

The development of the research met the national and international ethical precepts, being approved by the Research Ethics Committee of the State University of Londrina, according to Opinion No. 4,021,962 and CAAE 25811519.5.0000.5231.

## Results

The study included 213 students from technical Nursing courses, with a median age of 26 years old (IRQ:17); most were women (85.4%), single (56.3%), who lived with their family (90.1%), worked (62%) and did not use antidepressants (92.5%). Regarding the academic information, 71.4% were linked to private courses and 46.5% were enrolled in the last semesters of the course.

In the sample of this study, the Cronbach’s alpha values were α=0.813 for MBI, α=0.836 for ESS and α=0.704 for PSQI, respectively. Table 1 shows that 4.7% of the students had an indication of burnout syndrome; that 34.7% presented excessive daytime sleepiness and that 58.7% had poor sleep quality.

**Table 1:** Frequencies of the Maslach Burnout Inventory - Student Survey, Epworth Sleepiness Scale and Pittsburg Sleep Quality Index presented by the technical-level Nursing students (n=213). Londrina, PR, Brazil, 2020

Variables	Coding	Absolute frequency	Relative frequency
Emotional exhaustion	Low	111	52.1
	High	102	47.9
Depersonalization	Low	166	77.9
	High	47	22.1
Academic efficacy	Low	170	79.8
	High	43	20.2
Burnout syndrome	No	203	95.3
	Yes	10	4.7
Excessive daytime sleepiness	No	139	65.3
	Yes	74	34.7
Sleep quality	Good	51	23.9
	Poor	125	58.7
	Sleep disorder	37	17.4

Table 2 allows verifying that the participants with excessive daytime sleepiness had 5.714 times the chance of high emotional exhaustion when compared to those who were not classified as having this type of sleepiness. Likewise, all the PQSI components and poorer sleep quality significantly increased the chances of high emotional exhaustion.

**Table 2:** Association of emotional exhaustion with excessive daytime sleepiness and sleep quality among technical-level Nursing students (n=213). Londrina, PR, Brazil, 2020

Independent variables	p-value	odds ratio unadjusted* (95% Confidence Interval)	p-value	odds ratio adjusted† (95% Confidence Interval)
Excessive daytime sleepiness	**<0.001**	6.178(3.261-11.702)	**<0.001**	5.714(2.918-11.187)
Gender			0.029	0.354(0.140-0.898)
Age			0.053	0.966(0.932-1.000)
Work			0.621	1.186(0.604-2.328)
Use of antidepressants			0.180	0.437(0.130-1.466)
Subjective sleep quality	**0.013**	1.480(1.085-2.019)	**0.028**	1.435(1.039-1.981)
Gender			0.023	0.371(0.158-0.872)
Age			0.003	0.952(0.921-0.983)
Work			0.082	1.744(0.931-3.265)
Use of antidepressants			0.240	0.516(0.171-1.555)
Sleep latency	**<0.001**	2.137(1.490-3.065)	**<0.001**	2.160(1.467-3.183)
Gender			0.044	0.409(0.172-0.976)
Age			0.010	0.957(0.925-0.990)
Work			0.034	2.033(1.056-3.913)
Use of antidepressants			0.113	0.401(0.129-1.242)
Sleep duration	**0.006**	1.380(1.097-1.736)	**0.002**	1.513(1.172-1.955)
Gender			0.004	0.265(0.108-0.651)
Age			0.001	0.947(0.915-0.979)
Work			0.304	1.401(0.737-2.666)
Use of antidepressants			0.153	0.437(0.140-1.360)
Habitual sleep efficiency	**0.014**	1.543(1.093-2.177)	**0.019**	1.541(1.074-2.210)
Gender			0.020	0.361(0.153-0.852)
Age			0.003	0.950(0.920-0.982)
Work			0.085	1.749(0.926-3.303)
Use of antidepressants			0.167	0.444(0.140-1.405)
Sleep disorders	**<0.001**	3.986(1.919-8.279)	**<0.001**	4.463(2.026-9.829)
Gender			0.010	0.312(0.129-0.755)
Age			0.005	0.952(0.920-0.986)
Work			0.133	1.653(0.858-3.184)
Use of antidepressants			0.057	0.313(0.094-1.038)
Use of sleep medications	0.116	1.200(0.956-1.508)	0.080	1.246(0.974-1.594)
Gender			0.017	0.353(0.150-0.828)
Age			0.002	0.949(0.918-0.981)
Work			0.132	1.618(0.865-3.028)
Use of antidepressants			0.109	0.391(0.124-1.231)
Daytime dysfunction	**<0.001**	4.407(2.741-7.087)	**<0.001**	4.401(2.660-7.282)
Gender			0.043	0.375(0.145-0.969)
Age			0.085	0.969(0.934-1.004)
Work			0.123	1.721(0.863-3.428)
Use of antidepressants			0.068	0.304(0.085-1.094)
Sleep quality				
Good	**<0.001**		**<0.001**	
Poor	**0.001**	3.466(1.631-7.365)	**<0.001**	3.542(1.622-7.735)
Sleep disorders	**<0.001**	15.065(5.212-43.543)	**<0.001**	17.065(5.548-52.490)
Gender			0.008	0.293(0.117-0.731)
Age			0.009	0.954(0.921-0.988)
Work			0.107	1.740(0.886-3.417)
Use of antidepressants			0.086	0.355(0.109-1.157)

* Univariate binary logistic regression;

† Multiple binary logistic regression; Hosmer-Lemeshow of each model: 0.847, 0.109, 0.853, 0.322, 0.721, 0.523, 0.242, 0.139, 0.148; Nagelkerke *R Square* of each model: 0.253, 0.124, 0.189, 0.156, 0.130, 0.193, 0.113, 0.318, 0.263, respectively

There were increased chances of high depersonalization among students with excessive daytime sleepiness (OR: 4.259) and with a worse perception of subjective sleep quality (OR: 1.553), sleep latency (OR: 1,682), sleep disturbances (OR: 4.218), daytime dysfunction (OR: 2.452) and with sleep disorders (OR: 6.029) (Table 3).

**Table 3:** Association of depersonalization with excessive daytime sleepiness and sleep quality among technical-level Nursing students (n=213). Londrina, PR, Brazil, 2020

Independent variables	p-value	odds ratio unadjusted* (95% Confidence Interval)	p-value	odds ratio adjusted† (95% Confidence Interval)
Excessive daytime sleepiness	**0.001**	3.291(1.678-6.456)	**<0.001**	4.259(2.037-8.905)
Gender			0.640	1.265(0.473-3.383)
Age			0.268	1.022(0.983-1.062)
Work			0.033	0.436(0.203-0.936)
Use of antidepressants			0.282	0.418(0.086-2.045)
Subjective sleep quality	**0.025**	1.541(1.055-2.250)	**0.030**	1.533(1.043-2.252)
Gender			0.718	1.191(0.461-3.080)
Age			0.767	1.006(0.969-1.043)
Work			0.276	0.673(0.330-1.372)
Use of antidepressants			0.327	0.461(0.098-2.166)
Sleep latency	**0.019**	1.637(1.085-2.471)	**0.017**	1.682(1.096-2.583)
Gender			0.643	1.253(0.483-3.249)
Age			0.630	1.009(0.972-1.047)
Work			0.339	0.704(0.343-1.445)
Use of antidepressants			0.240	0.394(0.083-1.866)
Sleep duration	0.100	1.248(0.958-1.624)	0.050	1.317(1.000-1.734)
Gender			0.922	0.953(0.364-2.492)
Age			0.912	1.002(0.966-1.039)
Work			0.121	0.562(0.271-1.164)
Use of antidepressants			0.289	0.434(0.093-2.030)
Habitual sleep efficiency	0.058	1.397(0.988-1.973)	0.053	1.421(0.995-2.030)
Gender			0.745	1.170(0.455-3.006)
Age			0.803	1.005(0.969-1.042)
Work			0.248	0.657(0.322-1.341)
Use of antidepressants			0.277	0.423(0.089-1.995)
Sleep disorders	**0.002**	3.324(1.537-7.186)	**0.001**	4.218(1.803-9.870)
Gender			0.797	1.135(0.433-2.971)
Age			0.537	1.012(0.974-1.051)
Work			0.112	0.546(0.259-1.151)
Use of antidepressants			0.129	0.274(0.052-1.460)
Use of sleep medications	0.671	1.060(0.809-1.389)	0.428	1.121(0.845-1.488)
Gender			0.806	1.125(0.440-2.873)
Age			0.909	1.002(0.966-1.039)
Work			0.197	0.625(0.307-1.276)
Use of antidepressants			0.266	0.409(0.085-1.977)
Daytime dysfunction	**0.001**	2.177(1.360-3.485)	**0.001**	2.452(1.471-4.088)
Gender			0.561	1.336(0.504-3.540)
Age			0.284	1.022(0.982-1.063)
Work			0.154	0.588(0.284-1.220)
Use of antidepressants			0.253	0.402(0.084-1.920)
Sleep quality				
Good	0.005		0.003	
Poor	0.198	1.875(0.719-4.887)	0.155	2.023(0.765-5.347)
Sleep disorders	**0.002**	5.357(1.821-15.762)	**0.001**	6.029(1.990-18.269)
Gender			0.804	1.130(0.430-2.966)
Age			0.582	1.011(0.973-1.050)
Work			0.182	0.609(0.294-1.263)
Use of antidepressants			0.227	0.380(0.079-1.826)

* Univariate binary logistic regression;

† Multiple binary logistic regression; Hosmer-Lemeshow of each model: 0.571, 0.188, 0.893, 0.618, 0.329, 0.835, 0.687, 0.819, 0.474; Nagelkerke *R Square* of each model: 0.126, 0.053, 0.060, 0.046, 0.044, 0.106, 0.023, 0.108, 0.100, respectively

Table 4 shows that the students with high habitual sleep efficiency had 1.552 chances of low efficiency in their studies. Those with sleep disorders had 4.083 times the chance of low academic efficacy when compared to those with good sleep quality.

**Table 4:** Association of low academic efficacy with excessive daytime sleepiness and sleep quality among technical-level Nursing students (n=213). Londrina, PR, Brazil, 2020

Independent variables	p-value	odds ratio unadjusted* (95% Confidence Interval)	p-value	odds ratio adjusted† (95% Confidence Interval)
Excessive daytime sleepiness	0.845	1.073(0.529-2.174)	0.865	1.067(0.503-2.266)
Gender			0.025	2.680(1.132-6.347)
Age			0.412	0.983(0.943-1.024)
Work			0.443	0.742(0.346-1.591)
Use of antidepressants			0.475	0.571(0.123-2.655)
Subjective sleep quality	0.133	1.342(0.914-1.970)	0.134	1.358(0.910-2.027)
Gender			0.019	2.821(1.182-6.734)
Age			0.451	0.985(0.946-1.025)
Working			0.524	0.784(0.371-1.657)
Use of antidepressants			0.477	0.571(0.122-2.675)
Sleep latency	0.072	1.468(0.966-2.231)	0.054	1.543(0.992-2.399)
Gender			0.014	3.005(1.249-7.231)
Age			0.527	0.987(0.948-1.028)
Work			0.633	0.831(0.389-1.776)
Use of antidepressants			0.375	0.493(0.104-2.348)
Sleep duration	0.053	1.308(0.996-1.716)	0.076	1.295(0.973-1.723)
Gender			0.060	2.324(0.964-5.602)
Age			0.390	0.982(0.944-1.023)
Work			0.309	0.673(0.314-1.444)
Use of antidepressants			0.413	0.522(0.110-2.471)
Habitual sleep efficiency	**0.022**	1.503(1.060-2.130)	**0.018**	1.552(1.077-2.236)
Gender			0.018	2.883(1.202-6.914)
Age			0.490	0.986(0.947-1.027)
Work			0.508	0.775(0.364-1.650)
Use of antidepressants			0.360	0.477(0.098-2.330)
Sleep disorders	0.180	1.664(0.790-3.505)	0.181	1.729(0.775-3.858)
Gender			0.026	2.660(1.122-6.305)
Age			0.486	0.986(0.947-1.026)
Work			0.387	0.717(0.337-1.525)
Use of antidepressants			0.358	0.474(0.097-2.328)
Use of sleep medications	0.207	1.193(0.907-1.568)	0.115	1.265(0.944-1.694)
Gender			0.024	2.719(1.142-6.477)
Age			0.399	0.983(0.944-1.023)
Work			0.379	0.712(0.335-1.517)
Use of antidepressants			0.288	0.419(0.084-2.086)
Daytime dysfunction	0.485	1.179(0.742-1.874)	0.506	1.182(0.722-1.933)
Gender			0.022	2.753(1.157-6.552)
Age			0.487	0.985(0.946-1.027)
Work			0.442	0.746(0.354-1.574)
Use of antidepressants			0.449	0.550(0.117-2.583)
Sleep quality				
Good	0.009		0.009	
Poor	0.614	1.269(0.503-3.201)	0.706	1.201(0.465-3.100)
Sleep disorders	**0.009**	4.000(1.411-11.336)	**0.011**	4.083(1.386-12.030)
Gender			0.021	2.859(1.175-6.954)
Age			0.560	0.988(0.948-1.029)
Work			0.451	0.745(0.346-1.602)
Use of antidepressants			0.338	0.455(0.091-2.277)

* Univariate binary logistic regression;

† Multiple binary logistic regression; Hosmer-Lemeshow of each model: 0.196, 0.942, 0.380, 0.313, 0.188, 0.876, 0.281, 0.103, 0.986; Nagelkerke *R Square* of each model: 0.054, 0.071, 0.061, 0.076, 0.092, 0.067, 0.072, 0.057, 0.118, respectively

## Discussion

This study aimed at verifying the association of the burnout syndrome with daytime sleepiness and sleep quality among technical-level Nursing students and the results showed that excessive daytime sleepiness, significantly, increased the chances of high emotional exhaustion and high depersonalization. Sleep disorders were associated with all dimensions of the syndrome.

The characterization of the participants indicated young, single women who live with their families, facts that are similar to the profile of students attending nursing technician and assistant courses at the national level^(11)^. Considering the proportions of the investigated sample that were female (85.4%), married (43.7%), who worked (62%) and that the household activities are culturally and historically attributed to them, a number of studies identified that, when reconciling multiple activities, women are more likely to suffer mental wear out^(24)^, as well as sleep deprivation, deficit and disorders^(18,25)^.

The proportions of the burnout syndrome and the dimensions of emotional exhaustion, depersonalization and low academic efficacy obtained in this study (4.7%; 47.9%; 22.1% and 20.2%) were lower when compared to a Brazilian study with Nursing students (10.5%; 76.3%; 31.6% and 21.1%)^(4)^. Regardless of the rates, psychological distress must be prevented as it is associated with decreased academic performance^(19)^ and with the repercussions for the students’ health.

Excessive daytime sleepiness is frequent in students, with prevalence varying from 24.6% to 57.4%. In this study, the prevalence of excessive daytime sleepiness was 34.7%, consistent with the values found in other studies conducted with medical students from Morocco (36.6%)^(19)^ and China (24.6%)^(20)^ and with Nursing students from Indonesia (28.8%)^(17)^, but much lower when compared to the prevalence of excessive daytime sleepiness presented by Nursing students from Oman (57.4%)^(21)^. Poor sleep quality occurred with 58.7% of the participants in this study, being consistent with the study among Moroccan medical students (58.2%)^(19)^ and lower than in the research with Indonesian (66.0%)^(18)^ and Brazilian (67.0%) Nursing students^(22)^.

Sleep is a condition of periodic rest of the body and nervous system, fundamental for memory consolidation, as it favors the processing of new information. As such, it is crucial to the learning, performance and health of students in any area or level; its deprivation, even if partial, exerts a negative effect on learning^(26-27)^ and is associated with low academic performance^(19)^.

Sleep restriction is related to a series of health effects, such as increased mortality and decreased renal function^(28)^. Women who sleep less than 6 hours a day experience more depressive, stress and anxiety symptoms, due to the decline in the cognitive function, which results in impaired judgment and increased compulsion, exhaustion and despair^(29)^. In this sense, a systematic review identified that, among students, sleep quality and sleep hygiene are strong predictors of depression or depressive symptoms, as students with poor sleep quality are less likely to use adaptive coping strategies and have greater difficulty in diverting attention from negative stimuli^(30)^. In addition, long (>60 minutes) and regular daytime naps increased the risk of Parkinson’s disease^(31)^, type 2 diabetes mellitus, cardiometabolic risk^(32)^ and cardiovascular diseases^(33)^, as sleeping during the day is considered a complement to the normal sleep period, not reaching some of its physiological phases, in which restorative and protective benefits occur^(9)^.

The high prevalence values identified and the numerous consequences indicate the need for actions to deal with these problems, such as sleep hygiene programs and healthy sleep habits among the students^(21)^.

In this study, it was verified that excessive daytime sleepiness, all the PQSI components, and poor sleep quality significantly increased the chances of high emotional exhaustion. This burnout syndrome dimension is the first to emerge and shows exhaustion due to the insufficiency of psychological mechanisms to face the numerous study demands, necessary for training^(4)^.

Excessive daytime sleepiness is a chronic symptom arising from poor sleep quality, which is reflected in an inability to stay awake and/or alert during the day, causing harms in people’s physical, psychic and mental areas^(11)^. The students who are unable to concentrate on daily activities because of sleepiness may have an increased perception of activity overload and, consequently, an increase in exhaustion. In this sense, students who sleep enough hours daily have higher levels of psychological health^(30)^ and, on the other hand, poor sleep quality favors the emergence of emotional exhaustion^(34)^.

The association identified in this study between the worse perceptions of the sleep quality components and emotional exhaustion can be explained by the need for a third shift devoted to studying, as it is necessary to reconcile family life, household chores, work and studies. Such being the case, fundamental aspects related to the reduction of stress, anxiety and exhaustion, such as sleeping and eating habits, social life and leisure, are relegated due to studying off-shift^(35)^. It is believed that working and studying can become a high emotional burden and, often, there is no institutional support culture of for workers who study, impacting on the amount of sleep and on the use of psychoactive substances to regulate the awake-sleep cycle, i.e., stimulants to interrupt daytime sleepiness and sedatives to reverse such effects. However, both are associated with poor sleep quality and psychological disorders^(36)^.

Within the burnout syndrome *continuum*, depersonalization results from emotional exhaustion, that is, a way of dealing with the burden of the activities, showing negative attitudes towards the colleagues and the study, such as coldness and indifference^(6)^. It was verified that there were increased chances for high depersonalization among the students with excessive daytime sleepiness and worse perception of subjective sleep quality, sleep latency, sleep disorders, daytime dysfunction and sleep disorders. A study carried out in the United Arab Emirates with university students confirmed the association of depersonalization with poor sleep quality and daytime naps. The authors assert that dissociative experiences are enhanced by sleep quality, especially sleep disorders, due to the difficulty in regulating and controlling the consciousness states that lead to cognitive disinhibition^(37)^.

Inefficient sleep and the presence of sleep disorders were associated with low academic efficacy, which is an interdependent dimension of the teaching process, as it results from the difficulty in recognizing, valuing and obtaining results, generating feelings of insufficiency, powerlessness, insecurity, low self-esteem and inferiority. Low scores in this dimension were related to the decrease in the feelings of competence with the performance of a professional activity, among university students^(38)^.

Although the study achieved its objective, it had limitations due to the online data collection format required due to the covid-19 pandemic, as these individuals may not access their email addresses frequently. Another limit concerns the year of data collection, in which exhaustion may be exacerbated^(39)^. The cross-sectional design precludes adequately assessing causal relationships and it should be noted that the study was carried out in four schools from a single city, preventing generalization of the results. It is necessary to consider the scarcity of studies with students from professional courses in Nursing, being necessary to discuss the results with studies involving students from other training levels.

Despite these limits, this study advances in knowledge by investigating a population whose training is essential for the maintenance of the health services. It also contributes to the discussion of the theme, regarding the influence of sleep quality on the burnout syndrome, advancing in presenting the association of excessive daytime sleepiness and poor sleep quality, especially in the assessment of its components in relation to each dimension of the syndrome.

The results indicate the need to adopt individual and collective strategies, such as time management between studies and personal and professional life, so that pleasurable activities that produce physical and mental well-being are not neglected. Managers and professors of technical Nursing courses need to identify changeable aspects in the curricula that generate overload, as well as implement institutional policies and services for the well-being of their students. In this sense, new studies should address such aspects.

## Conclusion

The study hypothesis was confirmed, as high levels of the emotional exhaustion and depersonalization dimensions were associated with excessive daytime sleepiness and poor sleep quality in students of the technical Nursing course. Increased chances for the burnout syndrome were found among the students with sleep disorders, even after adjusting for the gender, age, work and depression covariates.

Such findings indicate the importance of students at this educational level having good sleep hygiene. The institutions that do not offer psychosocial support programs should plan and implement them, with a view to promoting the health of their students.
